# Detecting the Mechanism behind the Transition from Fixed Two-Dimensional Patterned Sika Deer (*Cervus nippon*) Dermal Papilla Cells to Three-Dimensional Pattern

**DOI:** 10.3390/ijms22094715

**Published:** 2021-04-29

**Authors:** Guanning Wei, Hongmei Sun, Haijun Wei, Tao Qin, Yifeng Yang, Xiaohong Xu, Shoujing Zhao

**Affiliations:** 1 School of Life Sciences, Jilin University, Changchun 130012, China; weign16@mails.jlu.edu.cn; 2 Institute of Special Wild Economic Animals and Plants, Chinese Academy of Agricultural Sciences, 4899 Juye Street, Changchun 130112, China; weihaijun@caas.cn (H.W.); yangyifeng@caas.cn (Y.Y.); 3 School of Ecology and Environment, Northwestern Polytechnical University, Xi’an 710072, China; wqqt@mail.nwpu.edu.cn; 4 Laboratory of Infectious Diseases, College of Veterinary Medicine, Jilin University, Changchun 130062, China; xuxiaohong@jlu.edu.cn

**Keywords:** sika deer, dermal papilla cell, 3D pattern, 2D pattern, hair inducing capability

## Abstract

The hair follicle dermal papilla is critical for hair generation and de novo regeneration. When cultured in vitro, dermal papilla cells from different species demonstrate two distinguishable growth patterns under the conventional culture condition: a self-aggregative three dimensional spheroidal (3D) cell pattern and a two dimensional (2D) monolayer cell pattern, correlating with different hair inducing properties. Whether the loss of self-aggregative behavior relates to species-specific differences or the improper culture condition remains unclear. Can the fixed 2D patterned dermal papilla cells recover the self-aggregative behavior and 3D pattern also remains undetected. Here, we successfully constructed the two growth patterns using sika deer (*Cervus nippon*) dermal papilla cells and proved it was the culture condition that determined the dermal papilla growth pattern. The two growth patterns could transit mutually as the culture condition was exchanged. The fixed 2D patterned sika deer dermal papilla cells could recover the self-aggregative behavior and transit back to 3D pattern, accompanied by the restoration of hair inducing capability when the culture condition was changed. In addition, the global gene expressions during the transition from 2D pattern to 3D pattern were compared to detect the potential regulating genes and pathways involved in the recovery of 3D pattern and hair inducing capability.

## 1. Introduction

Dermal papilla (DP) is a spherical or pear-shaped specialized mesenchymal condensation that is embedded in the hair bulb at the base of the follicle, enclosed by epithelial matrix keratinocytes [1]. The transplanting experiments of postnatal DP from various origins, such as feathers, pelage, and hairs, proved DP plays a key role in inducing hair follicle neogenesis in both follicular and non-hair-bearing skin [2,3,4,5]. In the process of hair generation and regeneration, DP instructs the keratinocytes to constitute the hair shaft and inner root sheath during the anagen phase [6,7,8]. The structure of DP is relatively simple, consisting of a predominant single cell type: DP cells [9]. In vitro, DP cells from different species demonstrate two distinctive patterns under conventional serum containing culture conditions: two dimensional (2D) monolayer polygonal pattern and three dimensional (3D) spheroidal pattern. In reported assays, DP cells from ovine wool follicles [10] and rodent vibrissa follicles [11,12] can spontaneously aggregate, adopting embryonic-like properties, and finally, form a papilla condensate-like 3D spheroidal clumps. The 3D patterned DP cells maintain trichogenicity [9,13,14,15,16,17,18] and can retain this inductivity in much later passages [9,11]. However, human [9,19,20] and canine DP cells [21,22] show striking different growth patterns under the same culture conditions. They maintain 2D growth pattern, which normally loses the ability to induce hair follicles after a few passages. Similarly, sika deer (*Cervus nippon*) DP cells do not aggregate spontaneously but maintain 2D growth patterns in the conventional medium [23]. Whether the loss of self-aggregative behavior relates to species-specific differences or improper culture conditions remains elusive. Previous studies tried to restore the lost key inductive properties of the fixed 2D-pattern human DP cells through adding additional inducing entities [11,18,24,25,26,27] or forcing the DP cells into a 3D state [9,19]. Whether the fixed 2D patterned DP cells can recover the self-aggregative behavior is unclear. In the present study, we proved it was the culture condition that determined sika deer DP cell growth pattern. We further detected the two growth patterns could transit mutually as the culture condition was exchanged. The fixed 2D patterned sika deer DP cells could recover the self-aggregative behavior and transit back to 3D pattern with restored hair inducing capability. To further reveal the mechanism behind the transition, an RNA-seq based transcriptome analysis was conducted to compare gene expression patterns between the fixed 2D patterned DP cells (UDP), the re-aggregating DP cells (MDP) and the restored 3D patterned DP cells (ADP). The candidate genes and regulating pathways contributing to the recovery of the 3D pattern and the hair inducing capability were predicted and verified through real-time PCR analysis.

## 2. Results

### 2.1. Sika Deer Dermal Papilla Cell Culture

**Figure 1:**
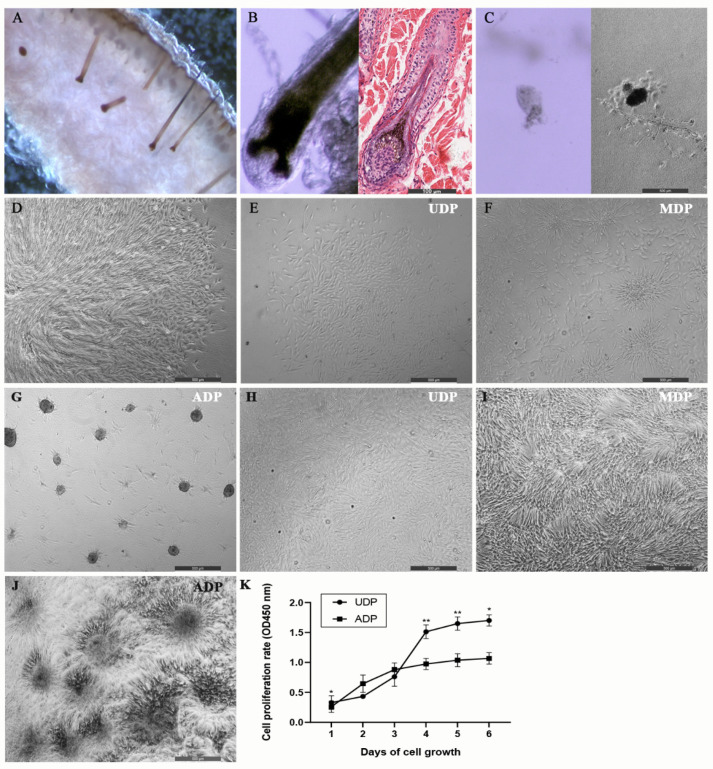
Sika deer dermal papilla isolation, cell culture, and cell proliferation test. (**A**) A slide of back skin is divided into strips around 0.5 cm in width and 1 cm in length along the longitudinal axis. (**B**) Bar = 100 µm. Isolated sika deer fur follicle (left) and longitudinal section of the sika deer fur follicle with HE staining (right). (**C**–**J**): bar = 500 µm. (**C**) Dissociative dermal papilla (DP) tissue sticks on the culture plate surface (left) and DP cells begin to migrate out from the attached DP tissue in primary culture (right). (**D**) The intact papillae eventually collapses when the dish is approaching 60% confluence in primary culture. (**E**) The sub-culture DP cells in 2D culture condition show 2D monolayer-growth pattern: flattened, polygonal, and fibroblastic morphology. (**F**) The sub-culture DP cells of the second passage begin to aggregate in 24–48 h when they are changed into 3D culture conditions. (**G**) The sub-culture DP cells of the second passage in 3D culture condition finally grow into 3D spheroidal pattern in 3 days (average diameter = 100–200 nm). (**H**) The sub-culture 2D patterned DP cells of the fifth passage kept 2D flattened morphology when they were cultured in 3D-culture condition for first passage. (**I**) The self-aggregative behavior begins to appear in the second passage after the medium transition, as the DP cells reach confluence under 3D-culture conditions. (**J**) The spheroidal appearance restores once DP cells attach the surface of the culture dish in the third passage under 3D-culture condition. (**K**) Comparison of cell proliferation between fixed 2D patterned DP cells (UDP) and restored 3D patterned DP cells (ADP) through paired t test. Cells (2000/well) are seeded and the proliferative rates were assessed using Cell Counting Kit-8 on the 1st, 2nd, 3rd, 4th, 5th, and 6th days. *Y*-axis represents the OD values at a wavelength of 450 nm. *X*-axis represents the period of cell growth. * *p* < 0.05, ** *p* < 0.01.

The DP tissues were isolated from individual furs from 15 sika deer for primary culture (Figure 1A,B). Spindle-shaped DP cells began to migrate out from the attached DP within 3–5 days (Figure 1C). The intact papillae eventually collapsed when the dish was approaching 60% confluence on the 8th day on average (Figure 1D). The sub-cultured DP cells of the first two generations in DMEM/deer serum showed monolayer growth-pattern with flattened, polygonal, and fibroblastic morphology and maintained the pattern for the rest passages (Figure 1E). Though in rare cases, some sub-cultured DP cell lines maintained the self-aggregative behavior and finally formed the 3D pattern after reaching confluence (less than 5%), the whole process is time consuming-normally lasting for one month and the aggregative behavior is soon lost in two generations.

### 2.2. Restoration of 3D-Pattern DP Cells from Fixed 2D Patterned DP Cells

After sub-culturing the selected three 2D patterned DP cell lines for two passages in 2D culture conditions, the 2D pattern was fixed. We first tried to change the 2D-culture condition into a 3D-culture condition for the fixed 2D patterned DP cells from different passages. The results showed the recovery duration of the self-aggregative behavior correlated with the fixed time in 2D pattern. The second or third 2D patterned DP cell passage normally recovered the self-aggregate behavior in 24–48 h (Figure 1F) and grew into 3D spheroids in 3 days (Figure 1G) after the 3D-culture condition was applied. The spheroids were 100–200 μm in diameter on average. However, the 2D patterned DP cells over the fifth passage did not recover the self-aggregative behavior in the first two passages when the 3D-culture condition was applied. They kept 2D flattened morphology but had faster proliferating speed in the first passage (Figure 1H) compared to those continually sub-cultured in 2D-culture conditions. The self-aggregative behavior appeared in the second passage after the 3D condition was applied, as the DP cells reached confluence (Figure 1I). Though 3D pattern could not form in most cases in this passage. Until the third passage after the 3D condition applied, the DP cells restored the 3D spheroidal appearance once they attached to the surface of the culture dish (Figure 1J). The restored 3D pattern would retain for the rest passages under 3D-culture condition. On the contrary, when the culture condition of fixed 3D patterned DP cells was changed into the 2D-culture condition, DP cells no matter how long they had been cultured in 3D-culture conditions would lose their behavior in one passage. The results of CCK-8 assay showed 2D-pattern DP cells had more rapid cell expansion than restored 3D-pattern DP cells in general (Figure 1K). When ADP began to aggregate on the first day and second day after seeding, their proliferation rates were slightly higher than UDP (*p* < 0.05). However, after the spheroids formed on the third day, the growth rate of ADP became moderate, whereas UDP reached their logarithmic phase, showed robust expansion. The growth rate of UDP kept significantly higher than those of ADP since the 4th day (*p* < 0.01).

### 2.3. Identification of Hair Inducing Activity through Specific Expression of CD133, SOX2, Versican, and ALPL on DP Cells

**Figure 2:**
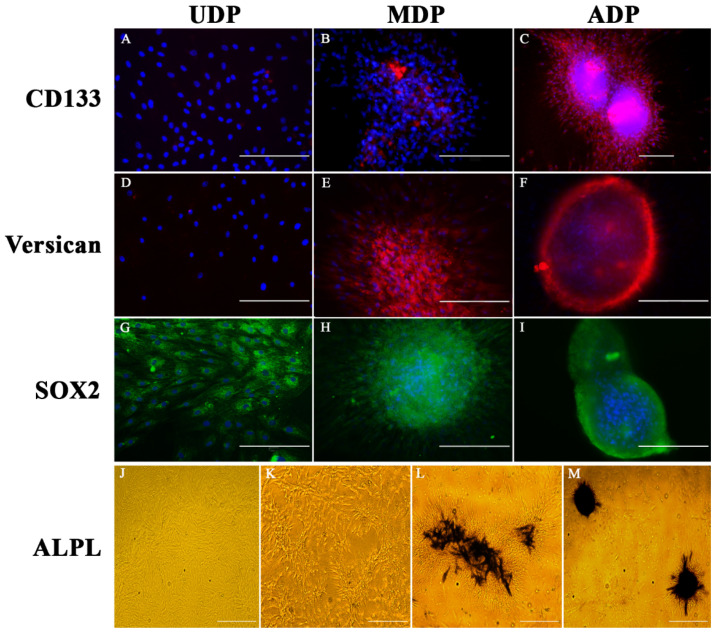
Identification of hair inducing capability of cultured DP cells. Bar = 200 µm. Comparison of immunofluorescence staining of CD133 (**A**–**C**), versican (**D**–**F**), SOX2 (**G**–**I**) and alkaline phosphatase (ALPL, **J**–**M**) on fixed 2D patterned DP cells (UDP), the re-aggregating DP cells (MDP) and restored 3D patterned DP cells (ADP). (**A**) UDP do not express CD133 (red) and DAPI (blue) marks all cell nuclei. (**B**) CD133 begins to express in MDP, but the expression intensity is weak (red). (**C**) ADP show strong CD133 expression intensity. (**D**) UDP do not express versican (red) and DAPI (blue) marks all cell nuclei. (**E**) Versican begins to express in MDP and the expression intensity is strong. (**F**) ADP continually show strong versican expression intensity. (**G**) UDP have strong expression intensity of SOX2 (green) and DAPI (blue) marks all cell nuclei. (**H**) The expression intensity of SOX2 (green) keeps strong in MDP. (**I**) ADP continually keep strong SOX2 expression (green). (**J**–**M**): bar = 500 µm. Comparison of Alkaline phosphatase (ALPL) staining during the process of ADP formation. (**J**) UDP do not show the ALPL staining. (**K**) DP cells do not express ALPL when they begin to aggregate. (**L**) MDP express strong expression of ALPL. (**M**) The ALPL expression reaches highest intensity in ADP.

CD133 and versican are two important makers of hair follicle-inducing capability in vivo [16,28,29]. Our results showed UDP did not express CD133 (Figure 2A) or versican (Figure 2D). MDP became CD133/versican positive as the culture condition changed (Figure 2B,E), and CD133 showed much lower expression level compared to versican in MDP. Both markers showed a high expression intensity in ADP (Figure 2C,F). The results confirmed that UDP lost hair-inducing capability. When DP cells restored aggregation, their hair-inducing capability gradually restored and reached the strongest level when spheroids formed.

Sox2 is an important indicator of the stem cells which are important for tissue regeneration [30]. In pelage hair follicles, Sox2 also functions as specifying the hair types [28,31,32]. In postnatal skin, Sox2 is only expressed in the DP of guard/awl/auchene follicles, whereas DP of the zigzag follicle is Sox2 negative [32]. Our results showed UDPs were Sox2 positive (Figure 2G). When the culture condition was changed, MDP and ADP continually kept the strong SOX2 expression (Figure 2H–I). The findings confirmed the stem cell identity of the cultured DP cells. Unlike versican and CD133, Sox2 expression in DP was an intrinsic property, unrelated to morphological change or hair inducing capability.

Alkaline phosphatase (ALPL) activity correlates closely with DP trichogenicity, but its changing pattern was mainly studied on DP tissues during hair cycle [18,33,34]. Our study first visualized the change of ALPL staining pattern in DP cells. According to the results, UDP did not express ALPL (Figure 2J). When DP cells began to aggregate as the culture condition changed, they did not express ALPL (Figure 2K) until MDP phase (Figure 2L). The ALPL expression reached the highest intensity in ADP phase (Figure 2M). The results further confirmed UDP lost hair-inducing capability, which is restored as DP self-aggregation reformed.

### 2.4. Transcriptome Analysis Revealed Important Genes Contributing to Restored 3D Pattern

**Figure 3:**
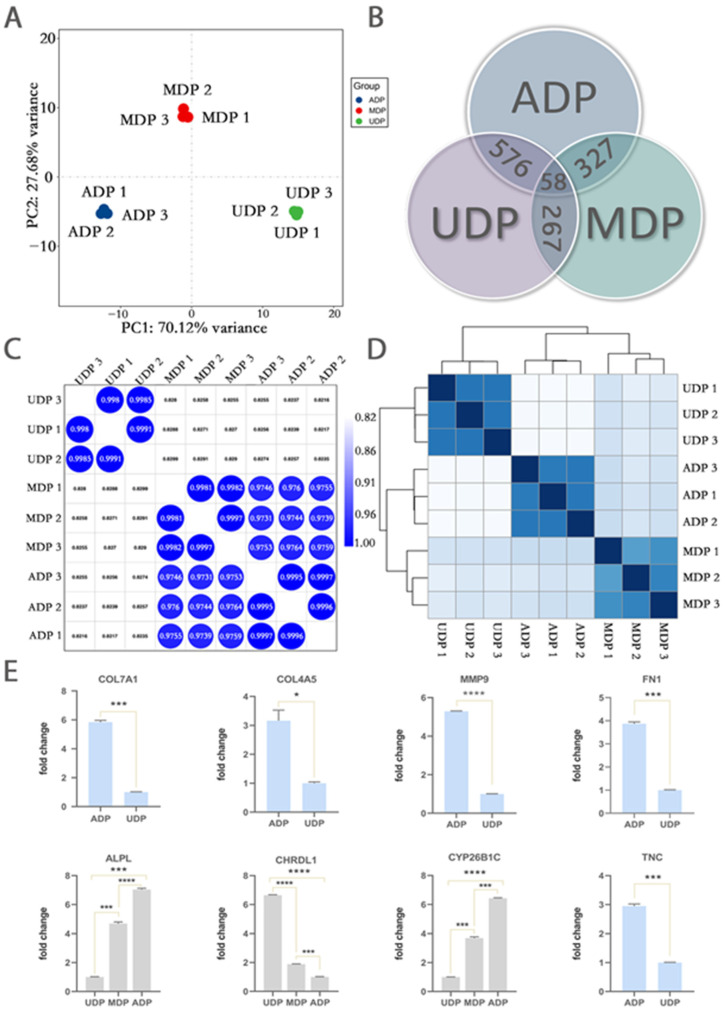
A general view of gene expression pattern and RT-PCR verification on selected DEGs. (**A**) The PCA analysis is performed on the transcript signatures of fixed 2D patterned DP cells (UDP), the re-aggregating DP cells (MDP), and restored 3D patterned DP cells (ADP). PC1 explained 70.12% of the overall variation. The distance between ADP and MDP is significantly shorter than that between MDP and UDP in PC1. (**B**) Venn analysis is performed on differentially expressed genes (DEGs) of UDP, MDP, and ADP. In total, 267 DEGs are expressed between MDP and UDP, 327 DEGs are expressed between MDP and ADP and 576 DEGs are expressed between ADP and UDP (|log2foldchange| ≥ 2, adjusted *p* value ≤ 0.05). Among them, 11 co-expressed DEGs in UDP, MDP, and ADP are further selected (|log2foldchange| ≥ 2, adjusted *p* value ≤ 0.05). (**C**) The correlation analysis is performed on the transcript signatures of UDP, MDP, and ADP. The average correlation coefficient between ADP and MDP is 0.98 (range: 0.97–0.98), whereas between ADP and UDP is 0.82 (range: 0.82–0.83) and between MDP and UDP is 0.83 (range: 0.826–0.829). (**D**) The hierarchical clustering analysis is performed on the transcript signatures of UDP, MDP, and ADP. Compared to UDP, MDP and ADP independently cluster together. (**E**) Verification of transcriptome results on the selected DEGs (RT-PCR, three individual wells/cell type) during the process of ADP re-formation, * *p* < 0.05, *** *p* < 0.001, **** *p* < 0.0001 (paired *t*-test).

To reveal the regulating genes in transition from UDP to ADP, we conducted RNA-seq analysis on UDP, MDP, and ADP. The hierarchical clustering was performed on the transcript signatures of UDP, MDP, and ADP. The results showed MDP and ADP independently clustered together (Figure 3D), whereas UDP from each donor were clustered. In addition, we calculated the correlation among UDP, MDP, and ADP (Figure 3C). The average correlation coefficient between ADP and MDP was 0.98 (range: 0.97–0.98), whereas between ADP and UDP it was 0.82 (range: 0.82–0.83), and between MDP and UDP, it was 0.83 (range: 0.826–0.829). Furthermore, in PCA analysis (Figure 3A), compared to PC2, PC1 explained 70.12% of the overall variation so that it could represent the overall trend. The distance between ADP and MDP was significantly shorter than that between MDP and UDP in PC1. The results of the three analyses together suggested compared to UDP, MDP showed a more similar gene expression pattern with ADP. The distinctive expressed gene patterns between UDP and MDP, ADP related to aggregative behavior.

**Figure 4:**
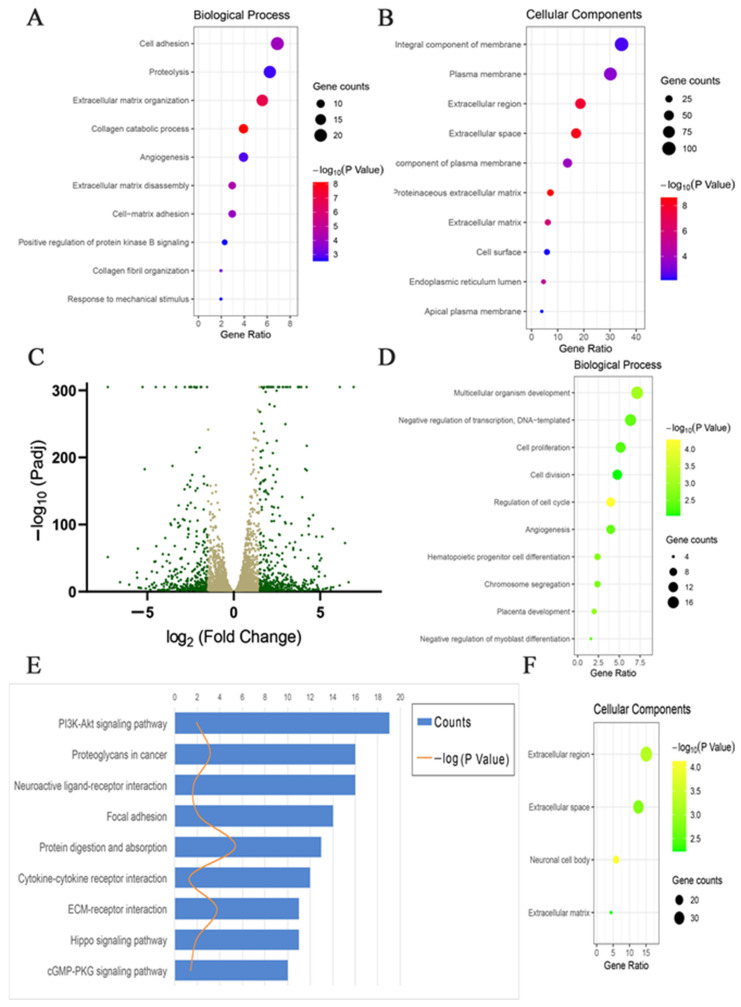
GO and KEGG analysis on DEGs between ADP and UDP. (**A**) GO analysis in BP is performed on the 214 up-regulated DEGs of ADP compared to UDP. The vertical axis represents the top 10 ranked GO terms according to the gene counts. The different colors from blue to red represent the −log 10 of adjusted *p* values (adjusted *p* values < 0.01). The horizontal axis represents gene ratio, which is the percentage of total DEGs in the given GO term. (**B**) GO analysis in CC is performed on the 214 up-regulated DEGs of ADP compared to UDP. The vertical axis represents the top 10 ranked GO terms according to the gene counts. The different colors from green to yellow represent the −log10 value of adjusted *p* values (adjusted *p* values < 0.01). The horizontal axis represents gene ratio, which is the percentage of total DEGs in the given GO term. (**C**) Volcano plot shows the expression level of DEGs between ADP and UDP. *X*-axis represents the log2 fold change values of DEGs between ADP and UDP. The values ≥2 or ≤−2 are marked by green color. *Y*-axis denotes –log10 value of adjusted *p* value (padj) of DEGs, padj < 0.01. (**D**) GO analysis in BP is performed on the 113 down-regulated DEGs of ADP compared to UDP. The vertical axis represents the top 10 ranked GO terms according to the gene counts. The different colors from blue to red represent the −log 10 of adjusted *p* values (adjusted *p* values < 0.01). The horizontal axis represents gene ratio, which is the percentage of total DEGs in the given GO term. (**E**) KEGG pathway analysis is performed on all DEGs between ADP and UDP. The blue bars of *X*-axis denote the gene counts of enriched KEGG pathway categories. The orange curve denotes the −log10 values of adjusted *p* values on enriched KEGG pathway categories, adjusted *p* value < 0.05. *Y*-axis represents the enriched KEGG pathway categories of all DEGs between ADP and UDP. (**F**) GO analysis in CC is performed on the 113 down-regulated DEGs of ADP compared to UDP. The vertical axis represents all 4 ranked GO terms according to the gene counts. The different colors from green to yellow represent the −log10 value of adjusted *p* values (adjusted *p* values < 0.01). The horizontal axis represents gene ratio, which is the percentage of total DEGs in the given GO term. GO, gene ontology; BP, biological process; CC, cellular component; ADP, restored 3D patterned DP cells; UDP, fixed 2D patterned DP cells; DEG: differentially expressed genes. Gene counts: the number of genes enriched in a GO term, represented by the different sizes of the circles.

The results of Venn analysis showed 267 DEGs between MDP and UDP, 327 DEGs between MDP and ADP, and 576 DEGs between ADP and UDP (Figure 3B, log2foldchange| ≥ 2, adjusted *p* value ≤ 0.05). Among them, 11 co-expressed DEGs in UDP, MDP, and ADP were further detected, including 6 gradually up-regulated genes and 5 gradually down-regulated genes (|log2foldchange| ≥ 2, adjusted *p* value ≤ 0.01, Table 1). In view of the similar expression between ADP and MDP, 576 DEGs between ADP and UDP were selected for further analysis. The DEGs included 317 up-regulated genes and 259 down-regulated genes of ADP compared to UDP (Figure 4C). The results of the biological process of GO enrichment analyses showed the up-regulated genes were mainly classified into the categories of cell adhesion, collagen catabolic process, extracellular matrix disassembly, cell-matrix adhesion, positive regulation of protein kinase B signaling and collagen fibril organization, etc. (*p* < 0.01, Figure 4A). The results of cellular component of GO enrichment analyses showed the up-regulated genes were mainly classified into the categories of integral component of membrane, plasma membrane, extracellular region, extracellular space, integral component of plasma membrane, proteinaceous extracellular matrix and extracellular matrix, etc. (*p* < 0.01, Figure 4B). Furthermore, the up-regulated genes of UDP compared to ADP enriched in biological process of GO terms were mainly related to cell proliferation, cell division, and regulation of cell cycle, etc. (*p* < 0.01, Figure 4D). The up-regulated genes of UDP compared to ADP enriched in cellular component of GO terms were mainly related to extracellular region, extracellular space, neuronal cell body, and extracellular matrix (*p* < 0.01, Figure 4F). The KEGG pathway analysis of all DEGs between ADP and UDP mainly included pathways in PI3K-Akt signaling pathway, proteoglycans in cancer, neuroactive ligand-receptor interaction, focal adhesion, protein digestion and absorption, cytokine-cytokine receptor interaction, ECM-receptor interaction, Hippo signaling pathway, and cGMP-PKG signaling pathway (*p* < 0.05, Figure 4E). The gene network of ECM-receptor interaction pathway was mapped to detect the specific regulating process (Supplementary Figure S1).

### 2.5. Real-Time PCR Analysis Verified Transcriptome Results

Five up-regulated DEGs of ADP compared to UDP (*COL4A5, COL7A1, MMP9, FN1, TNC*) were selected and validated by RT-PCR. The results showed that the expression levels of those genes were significantly higher in ADP than UDP (*p* < 0.01). In addition, three co-expressed DEGs from UDP, MDP, and ADP (*ALPL, Cyp26b1c, CHRDL1*) were validated by qRT-PCR as well. The expression levels of *ALPL* and *Cyp26b1c* significantly increased along with UPD, MDP, and ADP (*p* < 0.01) (Figure 3E). The expression level of *CHRDL1* significantly decreased along with UPD, MDP, and ADP (*p* < 0.01). The q-PCR results were the same as RNA-seq results.

## 3. Discussion

DP cells of several species, including human, maintain 2D pattern under conventional culture conditions, accompanied by the loss of hair inductive competence [9,35]. Whether the self-aggregative behavior is a species-specific capability and will the loss of the self-aggregative behavior be recovered remain unclear. In view of DP tissue as a specialized mesenchymal condensation [1], we considered using MSC medium to replace the conventional serum-containing culture medium for retaining the stemness maximumly. The results met our expectations and our study proved the sika deer DP cells in fixed 2D pattern could recover the spontaneous aggregation and 3D pattern with restored hair inductivity when the MSC medium was applied. It has been confirmed the expansion of mesenchymal stem cells strongly depends on the culture condition. Compared to the medium supplemented with 10–20% FBS, serum-free medium can better preserve the original morphology, function, and multipotentiality of the MSCs [36]. Our results suggested the spontaneous aggregation of DP cells was not a species-specific behavior, but closely correlated with the culture environment. It was more like to be lost under the stemness-suppression environments. When the suppression was released, the self-aggregative behavior would be recovered. It would be intriguing to try the same culture condition on human DP cells. Another interesting finding during the transition was the different proliferation rates between ADP and UDP. ADP showed a significantly lower proliferation compared to UDP according to the results of CCK8 test and transcriptome comparison. This finding was the same as the studies using DP cells from other species, which proved cultured aggregating DP cells had undergone comparatively few divisions and remained in low mitotic activity [20,37,38,39]. During the natural hair cycle, DP demonstrated the same low proliferative activity once established during embryonic development [40,41]. Our finding gave more proof that 3D-pattern DP cells more resembled natural DP behavior compared to 2D-pattern DP cells. The neogenesis of the hair follicles can be achieved by transplanting cultured ADP to the sub-epidermal space [24,42]. However, cell transplanting recipe for adult human hair-follicle neogenesis on a bald scalp has long been hampered by insufficient ADPs due to their low proliferation rate [43]. The rapid DP cell expansion in 2D pattern can theoretically be used for large-scale production of DP cells as seed bank. 3D-pattern DP cells can be induced once needed so that to resolve the insufficient ADPs in clinics.

For further detecting the mechanism behind this spontaneous transition, we performed global profiling of UDP and ADP. Previous studies proved the self-assembly of dispersed cells into 3D pattern or 2D pattern on biomaterials was switchable [44,45,46,47]. The formation of 3D pattern mainly depends on two factors: rate of cell collision and intercellular adhesion, following migration–collision–aggregation process [43,47,48,49,50]. The ultrastructural study [51] and microarray study on DP [52] both suggested cell adhesion contributed to DP aggregation. Another study found the formation of DP spheroids could be enhanced by specific coating proteins increased cell–cell adhesive strength while simultaneously maintaining high cell motility. On the contrary, the dispersed DP cells would not be able to aggregate if they cannot translocate to collide and adhere to each other [47]. Our results showed the largest amount of up-regulated genes of ADP compared to UDP was enriched in the GO term of cell adhesion, which gave more proof that cell adhesion played a vital role in DP cell aggregation. During natural hair development, the dispersal of condensates leads to papilla miniaturization and finally the disruption of hair-follicle morphogenesis [53]. Preventing the dispersal process through enhancing DP cell adhesion may offer a new strategy for treating androgenetic alopecia. Among the up-regulated genes in ADP relating to cell adhesion, *fibronectin* (*FN1*) may play an important role. *FN1* coded fibronectin is an adhesive glycoprotein, which is associated with cell adhesion and migration [54,55]. *FN1* locates in the extracellular matrix of DP from the embryonic stage [56,57]. Blocking of *FN1* leads to the interruption of epithelial-mesenchymal interaction and cell proliferation [58]. During the pelage hair cycle, *FN1* is prevalent in the DP during anagen but is lost in catagen and telogen [59]. In vitro, *FN1* coating is reported to significantly increase the number of DP spheroids through increasing DP cell adhesion and maintaining high cell motility simultaneously [47]. Our results showed the expression of *FN1* significantly increased in ADP compared to UDP. It may function as recruiting new cells (in vitro neighbor DP cells/in vivo connective tissue sheath cells) to migrate, collide and adhere to each other to form DP aggregation. *Tenascin* (*TNC*) is another important gene relating to cell adhesion, which encodes hexameric glycoprotein [60]. *TNC* refers to mesenchymal cell aggregation [61]. During the estimated gestational stage, it appears initially as focal deposits on the mesenchymal side of the dermal-epidermal junction during estimated gestational stage [58,60,62,63]. The follicle pre-germs develop subsequently at deposit sites. During the development of hair follicle *TNC* remains intensely in follicle basement membrane zone, interfollicular basement membrane zone, follicle health ECM and DP [64]. Our results suggested the *TNC* significantly up-regulated in ADP compared to UDP. It may contribute to promoting DP aggregation through increasing cell adhesion.

Furthermore, we are intrigued by detecting the molecular mechanism behind the restored hair inducing capability contributed by the 3D spheroidal pattern. The results of GO analysis in biological process and cellular component both suggested most of the categories enriched by up-regulated genes of ADP compared to UDP correlated with extracellular matrix (ECM) metabolism. This finding was consistent with a study on human DP spheres [9]. ECM serves an important role in tissue and organ morphogenesis and in the maintenance of cell and tissue structure and function [65]. In natural hair development, ECM is produced far before hair follicle morphogenesis by pre-follicular tissue. Early in the embryonic-fetal transition phase of gestational age, various ECM molecules are produced to concentrate beneath the basal lamina of the embryo, which cause the basement membrane to appear thickened and gradually form a broader, second sub-epidermal zone of dermis, referred to as compact mesenchyme. As the mesenchyme cells begin to aggregate to form condensed mesenchyme- DP precursors, some of the ECM molecules distributed generally throughout the compact mesenchyme prior to follicle initiation become restricted in distribution to DP [57]. During the hair cycle, the volume of ECM changes periodically with DP cell numbers. At the onset of anagen, DP displays synthetic activity and becomes separated by increasing ECM along with increased DP cells mainly compensating by connective tissue sheath cells [66]. The volume of the ECM reaches the largest amounts at the end of anagen [66], then are progressively reduced by decreased DP cell numbers during catagen, and are finally poorly developed by minimal DP cells on telogen [13]. In a study of de novo hair generation, the sub-dermal injection of ADP initially formed a papillae condensate-like clump that synthesizes its own ECM [12]. ECM produced by DP is mainly related to direct matrix keratinocytes to differentiate into distinct follicular layers during the hair cycle [67]. Therefore, we can reasonably speculate that accumulated ECM would be the key step in restoring hair inducing capability of 3D-pattern DP cells. ECM is a complex mixture of matrix molecules, involving various metabolic processes. Previous studies identified several molecules of the ECM produced by DP, but they were detected individually [39]. Our study first offered a general view of ECM relevant genes up-regulated in 3D-pattern DP cells compared to 2D-pattern DP cells, including cell-matrix adhesion, extracellular matrix organization, extracellular matrix disassembly, collagen catabolic process, and collagen fibril organization. We additionally identified specific gene signaling pathways involving ECM metabolic processes, including ECM-receptor interaction, focal adhesion, and PI3K-Akt signaling pathway. The genes and signaling pathways may help to uncover the specific functions of ECM in hair restoring ability of 3D cultured DP cells.

Among the up-regulated ECM genes, genes from the collagen family and matrix *metalloproteinases (MMPs*) family took high proportion. Collagens play structural roles and contribute to mechanical properties, organization, and shape of tissues, whose superfamily comprises 28 members in vertebrates [68]. *Collagen I, III, IV* has been widely reported to express in DP [57,69]. Our results suggested the expressions of those collagen encoding genes were up-regulated in 3D-pattern DP cells compared to 2D-pattern DP cells. Besides, *COL5A3*, *COL7A1*, *COL11A2*, and *COL27A1* showed the same expression pattern. *COL7A1* encoding collagen VII can be assembled into anchoring fibrils [70,71,72]. *COL27A1* encoding collagen XXVII forms 10 nm nonstriated fibrils [73]. Different collagens can also be comprised into collagen fibrils based on their supramolecular assemblies and the cross-linking is tissue-specific [74]. GO results suggested the collagen fibrils formed in DP aggregation included *COL1A2* encoding collagen I, *COL3A1* encoding collagen III, *COL5A3* encoding collagen V and *COL11A2* encoding collagen XI. The assembled fibrils may undertake the mechanical properties that offer a stable environment for the interactions between cells and ECM. *MMPs* are the main group of enzymes responsible for the collagen degradation in ECM, involving in the processes of proteolysis, collagen catabolic process, and extracellular matrix disassembly [75,76]. We detected that *MMP9, MMP13 MMP15, MMP16*, and *MMP19* were up-regulated in ADP compared to UDP. Among them, *MMP-13* preferential cleaves collagen II [77], whereas collagen IV is the preferential substrate of *MMP-9* [78]. *MMP-15* and *MMP-16* are reported to increase cell adhesion [76].

We additionally identified co-expressed DEGs in UDP, MDP, and ADP, including *Cyp26b1, CHRDL1, ALPL*, and *ADAM8*. The expression of *Cyp26b1c* increased along with DP aggregation. *Cyp26b1c* is one of the retinoic acid (RA)-degrading enzymes of the cytochrome P450 26 subfamily, which is specifically expressed in the dermis surrounding the developing hair follicles [79,80] and DP [81]. Excess RA has been shown to induce catagen-like hair follicle regression [82,83]. The ablation of *Cyp26b1* in embryonic period leads to excessive endogenous RA, resulting in the arrest of hair follicle growth at the hair germ stage, which can be rescued by the normalization of RA level. The conditional deficiency of *Cyp26b1* in the dermis also leads to excessive endogenous RA resulting in decreasing hair follicle density and a specific effect on hair bending [81]. The up-regulated *Cyp26b1c* along with DP aggregation may function as degrading RA so that to promote follicle growth. The *ALPL* is another important gene that showed significantly increased expression during DP aggregation. The recovery of hair inductivity is accompanied by restoration of *ALPL* expression in DP cells [9,20,84,85]. In the pelage follicles, AP activity is constantly and uniformly detected in the DP during the hair cycle [33], whereas in vibrissal follicles it dynamically changes, peaking in the early anagen phase [34]. In cell culture, majority primary-cultured DP cells express ALPL during the first few days, but later they began to lose *ALPL* expression. By the second passage, <5% were *ALPL*-positive [18]. Our results of ALPL staining and transcriptome analysis both suggested sub-cultured 2D-pattern AP cells lost *ALPL* expression. When they were reprogrammed into 3D pattern, their *ALPL* expression recovered and reached the highest level as spheroids formed. The results suggested the reforming ALPL in 3D-pattern DP cells might be one of the important elements contributing to follicle generation. On the contrary, the expression of *CHRDL1* was decreased along with DP aggregation. *CHRDL1* encodes secreted glycoprotein Chordin-like 1 (x) [86]. *CHRDL1* is known as a common bone morphogenetic proteins (BMP) inhibitor, sequestering the BMP in the extracellular space [86,87,88]. The hair follicle bulb microenvironment is rich in BMPs that act on DP cells to improve hair-inducing activity. The declined expression of *CHRDL1* in DP aggregation may increase BMP signaling so that to improve hair-inducing activity. The *ALPL* expression is also highly specific to members of the BMP family [18]. The over-expression of BMP receptors is reported to lead to robust maintenance of AP activity in DP. The declined expression of *CHRDL1* may also contribute to the increasing level of *ALPL* through increase BMP signaling during DP aggregation.

To conclude, our study constructed two fixed growth patterns of sika deer DP cells and proved it was the culture condition that determined sika deer DP growth pattern. The two growth patterns could transit mutually as the culture condition was exchanged. The fixed 2D-pattern sika deer DP cells could recover the self-aggregative behavior and 3D pattern, accompanied by the restoration of hair inducing capacity when the appropriate culture condition was applied. We additionally offered a general view of genes and pathways regulating the transition process. Among them, cell adhesion relevant genes played important roles in the formation of aggregation. ECM metabolism relevant genes and regulating networks were essential for the restoration of hair-inducing capacity. These findings represent a significant advance on recovering DP cell function so that to lay the basis of cell transplanting therapy on hair regeneration.

## 4. Materials & Methods

### 4.1. Animals

The skin tissues were harvested through surgical operation on 15 farmed male sika deer (*Cervus nippon*), with ages ranging from 2–3 years old immediately after the anesthetization in summer. After shaving the furs, the skin was sterilized through 75% ethanol and iodine solution. A 1 × 2 cm^2^ [2] skin cut was made and placed into DMEM medium (11965092, Gibco, Grand Island, NY, USA) with 5× penicillin-streptomycin (03-031-5B, BI, Beit Haemek, Israel) solution, 4 °C, then taken back to the lab. The skin incision was surgically sewn up, coated with penicillin and streptomycin powder. Finally, the deer were relieved from anesthetization.

### 4.2. Dermal Papilla Isolation

After washing by three consecutive PBS washes, the skin was divided into strips around 0.5 cm in width and 1 cm in length along the longitudinal axis. The strips were put into the 60 mm Petri dishes (P5481, Sigma, Darmstadt, Germany) containing 0.2% (*w*/*v*) collagenase D (11088858001, Roche, Agawam, MA, USA) in DMEM medium (11965092, Gibco) with 2× penicillin-streptomycin solution (03-031-5B, BI), 37 °C for 2 h. The surgical microdissection was used for acquiring DP tissue. The hair follicles were separated first from other tissues using a 27G needle and ophthalmic forceps. A slight cut was made at approximately three-quarters of the end bulb of the follicle for squeezing the DP out. After cutting through the stalk, an intact DP was transferred to a dish for primary culture.

### 4.3. Establishment of DP Cell Lines

**Figure 5:**
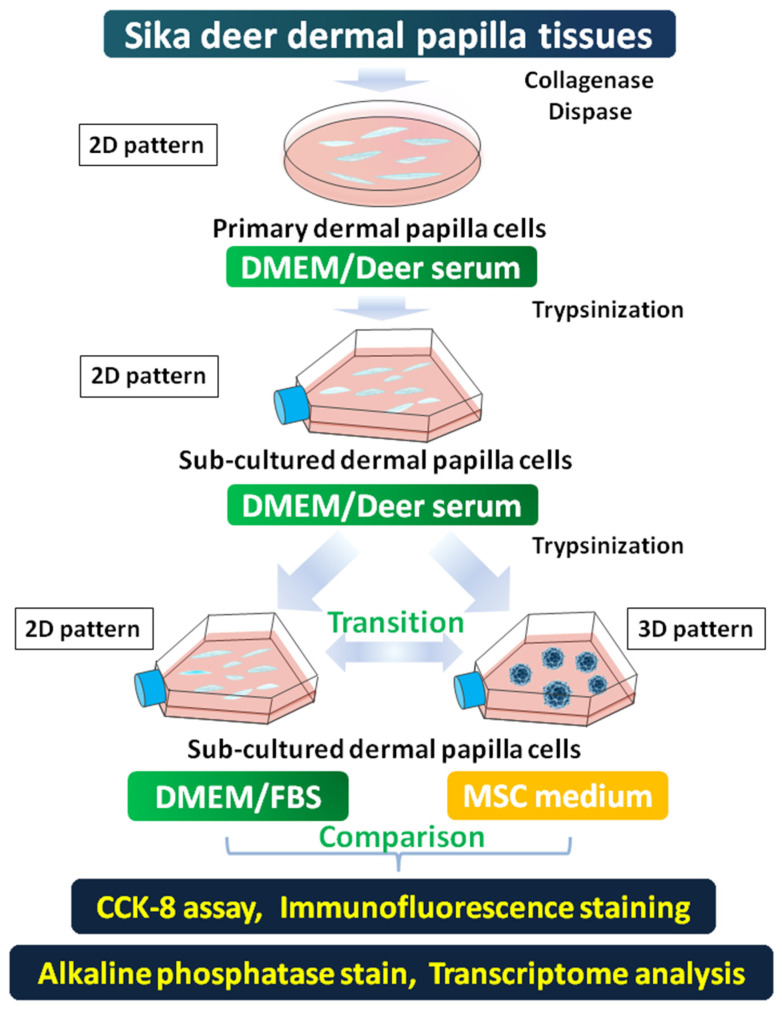
A schematic visualized the establishment of different DP cell growth patterns and the following analysis procedures.

The individual DP tissues were adhered to the base of the dish through a slight scratch using the needle. Around 6–10 DPs were cultured together in each 60 mm Petri dish (P5481, Sigma) containing 20% (*v*/*v*) in-house made sika deer serum in DMEM medium (11965092, Gibco), with 1× penicillin-streptomycin solution (03-034-1B, BI). After the 15 sika deer DP primary cell lines were established, the cells were sub-cultured for two generations for obtaining more DP cells. Specifically, after approaching 60% confluence, the primary DP cells were detached using 0.08% trypsin-EDTA and cultured in 25 flasks (CLS430372, Corning^®^, Canton, NY, USA), containing 10% (*v*/*v*) in-house made sika deer serum in DMEM medium (11965092, Gibco, 37 °C, 5% CO_2_). Half the amount of the cells from the 15 sub-cultured DP cell lines were preserved in liquid nitrogen and the rest were used for the pilot study. The formal experiments were conducted using three cell lines randomly selected from the 15 DP sub-cultured cell lines. All the cells used for the pilot study and the formal experiments were between passage two and eight. The DP cells in 2D pattern were established using DMEM medium (11965092, Gibco)/10% FBS (10099141, Gibco), with 1 × penicillin-streptomycin solution (03-034-1B, BI). The mesenchymal stem cell (MSC) medium (T310jv, Yuanpei, Shanghai, China) with 1× penicillin-streptomycin solution (03-034-1B, BI) was used for recovering the self-aggregative behavior and 3D pattern. A schematic was drawn to visualize the establishment of different DP cell growth patterns and the relevant analysis (Figure 5).

### 4.4. Cell Counting Kit-8 Assay

The comparison of cell proliferation rates between ADP and UDP was conducted using the Cell Counting Kit-8 (C0037, Beyotime, Shanghai, China) according to the manufacturer’s instructions. 2000 cells from each cell pattern were seeded into each well of a 96-well plate with a volume of 100 μL medium. Three biological replicates were performed for each type of cell. The 1st day, 2nd day, 3rd day, 4th day, 5th day, and 6th day were set as the test time points. After adding 10 μL CCK-8 reagent to each well, the plate was incubated for 2 h at 37 °C and 5% CO_2_. The absorbance of each well was measured at 450 nm and the optical density (OD) was determined via a microplate reader. A paired two-tailed t test was used to calculate the difference between samples and *p* value < 0.05 was considered as significant.

### 4.5. Immunofluorescence Staining

The cultured DP cells were fixed using 4% (*w*/*v*) paraformaldehyde fixation for 10 min. The cells were permeabilized for 5 min using PBS with 0.3% Triton X-100 (T8787, Sigma). Blocking was performed for 30 min using 3% (*w*/*v*) bovine serum albumin in PBS. The primary antibodies including rabbit anti-CD133 (1:500; Abcam, Cambridge, MA, USA), mouse anti-SOX2 (1:200; SantaCruz, Santa Cruz, CA, USA) and rabbit anti-Versican (1:500; Bioss) left overnight at 4 °C. The next day, the primary antibodies were washed off using three consecutive PBS washes. The secondary antibody, Alexafluor 594 goat anti-rabbit (1:1000; Thermo, Agawam, MA, USA)/Alexafluor 488 rabbit anti-mouse (1:1000; Thermo) was applied for 1 h at room temperature. The nuclei of cells were counterstained with DAPI (C1005, Beyotime) for 5 min at room temperature after removing the secondary antibody using three consecutive PBS washes. The coverslips were mounted using anti-fade reagent and the cells were visualized on a Zeiss Exciter fluorescent microscope. The specific information on all the antibodies used is listed in Supplementary Table S1.

### 4.6. Alkaline Phosphatase Stain

The cultured DP cells were fixed using 4% (*w*/*v*) paraformaldehyde fixation for 10 min and washed by PBS. The cells were incubated in BCIP/NBT staining solution (Beyotime, P0321) for 4 h in the dark at room temperature. The reaction was stopped by washing with PBS, and the cells were examined under a bright field microscope. Dark blue staining indicates positive signal for alkaline phosphatase stain (ALPL).

### 4.7. RNA Isolation and Real-Time PCR

The cells were briefly washed three times by PBS. Total RNA was isolated and purified using RNeasy mini kit 50 (151043752, Qiagen, Germantown, MD, USA), according to the manufacturer’s instructions. The RNA was quantified by measuring optical absorbance at 260 nm using Pro2000, Tecan, Switzerland and 200 ng was reverse transcribed cDNA using PrimeScript^TM^ RT reagent Kit (RR047A, Takara, Shiga, Japan) and oligo dT primers following the manufacturer’s instructions. Real-time PCR was performed using SYBR green I Master mix (4707516001, Roche) on an Applied Biosystems 7300 Real-Time PCR System. GAPDH were used in each reaction as a baseline control. Fold changes were calculated using the delta-delta CT algorithm, relative to GAPDH. A paired two-tailed t test was used to calculate the differences between samples and *p* value < 0.05 was considered as significant. Three biological replicates were performed for each gene of interest. GraphPad Prism ver. 8.2.0 (H. Motulsky, San Diego, CA, USA) was used to analyze data. The primer information is listed in Supplementary Table S2.

### 4.8. Transcriptome Analysis

Total RNA of UDP, MDP, and ADP were extracted using a Trizol reagent (Invitrogen Inc., Camarillo, CA, USA) according to the manufacturer’s procedure. RNA integrity was evaluated using the Agilent 2100 Bioanalyzer (Agilent Technologies, Santa Clara, CA, USA). The samples with RNA Integrity Number (RIN) ≥ 7 were subjected to the subsequent analysis. The libraries were constructed using TruSeq Stranded mRNA LTSample Prep Kit (Illumina, San Diego, CA, USA) according to the manufacturer’s instructions. Then these libraries were sequenced on the sequencing platform (Illumina HiSeq X ten, OEbiotechCo., Ltd., Shanghai, China) and 125 bp/150 bp paired-end reads were generated. Raw reads were processed using Trimmomatic [89]. The reads containing ploy-N and the low quality reads were removed to obtain the clean reads. The clean reads were then mapped to reference genome of Cervus elaphus hippelaphus using hisat2 [90]. The counts of the mapped reads were obtained by eXpress [91]. FPKM value of each transcript was calculated using bowtie2 [92].

### 4.9. Bioinformatics Analysis on Gene Expression Pattern and Differentially Expressed Genes

The gene expression patterns of UDP, MDP, and ADP were compared through hierarchical clustering analysis, Pearson correlation analysis, and PCA analysis. The hierarchical clustering analysis was performed used the hclust function of R package stat and visualized with heatmap 2 function of R package gplots [93]. The Pearson correlation analysis was performed with rcorr function of R package Hmisc [94] and visualized with corrplot R package [95]. The PCA analysis was performed using the R package prcomp and visualized by R package ggplot2 [96]. The differentially expressed genes (DEGs) were identified using the DESeq R packages [97] and visualized in vacano plot using GraphPad Prism 8 (ver. 8.2.0). The adjusted *p* value ≤ 0.05 and |log2FoldChange| ≥ 2 were set as the threshold for significantly differential expression. Venn analysis was used to calculate the numbers of DEGs between different types of DP cells by excel. The GO enrichment analysis of DEGs was performed using David v6.8 [98] (https://david.ncifcrf.gov/, accessed on 6 March 2020) and visualized using ggplot2 R package [96]. The KEGG pathway enrichment analysis of DEGs was performed using David v6.8 [98] and visualized using excel.

## Figures and Tables

**Table 1:** The 11 co-expressed DEGs ^†^ in UDP, MDP, and ADP ^‡^, including six gradually up-regulated genes and five down-regulated genes (|log2foldchange| ≥ 2, adjusted *p* value ≤ 0.01).

Gene ID	Gene Name	Gene Symbol	Log2FoldChange ADP vs. MDP	Log2FoldChange MDP vs. UDP
Celaphus_00014740	Alkaline phosphatase, tissue-nonspecific isozyme	*ALPL*	2.1	4.3
Celaphus_00011342	Liprin-alpha-2	*Ppfia2*	2.2	2.8
Celaphus_00005746	Cytochrome P450 26B1	*CYP26B1*	2.2	2.1
Celaphus_00011343	Liprin-alpha-4 (Fragment)	*Ppfia4*	2.7	2.1
Celaphus_00008669	Disintegrin and metalloproteinase domain-containing protein 8	*ADAM8*	3.7	2.1
Celaphus_00001834	SLAM family member 8	*SLAMF8*	2.13	2.12
Celaphus_00005340	Kelch-like protein 29	*KLHL29*	−4.2	−2.4
Celaphus_00012937	Solute carrier organic anion transporter family member 2A1	*Slco2a1*	−2.2	−2.5
Celaphus_00009791	Chordin-like protein 1	*CHRDL1*	−2.7	−2.6
Celaphus_00013117	Insulin-like growth factor II	*IGF2*	−5.8	−3.1
Celaphus_00001878	Platelet endothelial aggregation receptor 1	*PEAR1*	−3.8	−3.5

^†^ DEGs: differentially expressed genes; ^‡^ UDP: fixed 2D patterned DP cells, MDP: the re-aggregating DP cells, ADP: restored 3D patterned DP cells.

## Data Availability

Data is contained within the article or supplementary materials.

## Supplementary Materials

Supplementary materials can be found at https://www.mdpi.com/article/10.3390/ijms22094715/s1.
