# Supplementary material for: Detecting the Mechanism behind the Transition from Fixed Two-Dimensional Patterned Sika Deer (Cervus nippon) Dermal Papilla Cells to Three-Dimensional Pattern
Source: Int J Mol Sci. 2021 Apr 29;22(9):4715. doi: 10.3390/ijms22094715 (PMC8124381; doi:10.3390/ijms22094715)
Supplement: Supplementary file 1 [file ijms-22-04715-s001.zip › Supplementary Files/ijms-1128628-Supplementary Table S1.pdf]

Supplementary Table S1 Primers used in this study

Gene	Forward primer (5'-3')	Reverse primer (5'-3')	Product size (bp)
ALPL	TGATCCCACCATTCTTAGTGCTG	CTGAGCTTGTTCTCGCCAGTA	101
COL7A1	GGTCCCCAAGGTCTGTATCCTCA	CCTCAAGATGCTGAAGTCGTT	199
COL4A5	ATCTCCAGGTGATAGAGGACT	GAGGCCCTGAAACACCAGT	98
MMP9	GCCACCACCTCCAACTTCG	TGGATGCCCTGAACATCGTC	200
FN1	CTGAGACCCCAAGTCAACCC	TAAGCTGGCCCTCGTATACCAC	195
TNC	TCCACAGCCGGAGAACCTGA	GTTCGTTCAGCACCAGAGACGTT	176
CYP26B1	TTCTCCTTGCCCGTCGACT	CTTCAGCTCCTGCATGGTCA	198
CHRDL1	GGAGCACTCATGGATTCCCAAC	AGCACACACTCCACAATGCC	164
GAPDH	CATCGTCGCCATCAATGACCC	TGATGACGAGCTTCCCGTTC	122
