# Supplementary material for: Detecting the Mechanism behind the Transition from Fixed Two-Dimensional Patterned Sika Deer (Cervus nippon) Dermal Papilla Cells to Three-Dimensional Pattern
Source: Int J Mol Sci. 2021 Apr 29;22(9):4715. doi: 10.3390/ijms22094715 (PMC8124381; doi:10.3390/ijms22094715)
Supplement: Supplementary file 1 [file ijms-22-04715-s001.zip › Supplementary Files/ijms-1128628-Supplementary Table S2.pdf]

Supplementary Table S2 Antibodies used in immunofluorescence

Terms	Manufacturer and product code	Dilution ratio
Rabbit anti-CD133	Abcam; ab19898	1:500
Mouse anti-Sox	SantaCruz; sc-398254	1:200
Rabbit anti-Versican	Bioss; bs-2533R	1:500
DAPI	Beyotime; C1005	
Alkaline phosphatase	Beyotime; P0321	
Goat anti-rabbit (Alexa Fluor 594)	Thermo; A-11012	1:1000
Rabbit anti-mouse (Alexa Fluor 488)	Thermo; A-11059	1:1000
