# Supplementary figures and images for: Detecting the Mechanism behind the Transition from Fixed Two-Dimensional Patterned Sika Deer (Cervus nippon) Dermal Papilla Cells to Three-Dimensional Pattern
Source: Int J Mol Sci. 2021 Apr 29;22(9):4715. doi: 10.3390/ijms22094715 (PMC8124381; doi:10.3390/ijms22094715)

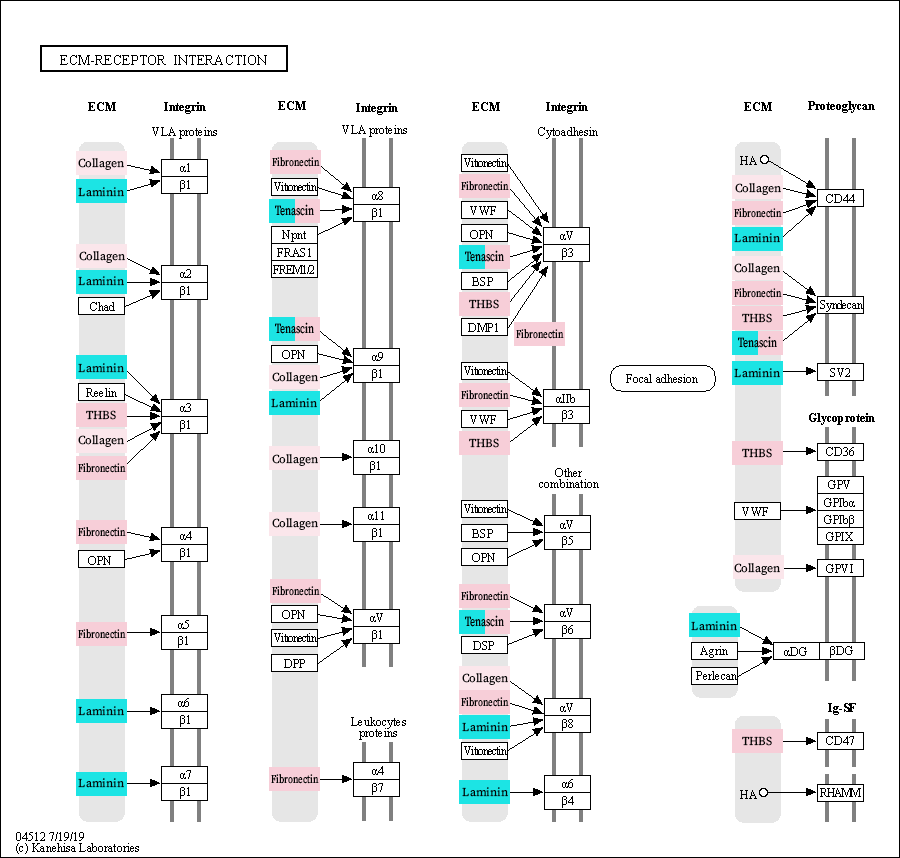

Supplement: Supplementary file 1 [file ijms-22-04715-s001.zip › Supplementary Files/ijms-1128628-Supplementary Figure S1.tif]
